# Valorization of *Cavia porcellus* By-Products via Ultrasound-Assisted Collagen Extraction: Optimization and Characterization

**DOI:** 10.3390/foods14203542

**Published:** 2025-10-17

**Authors:** Gussieff Lino Santos, Milady Esteban Valenzuela, Greta Hinostroza-Quiñonez, Omar Flores Ramos, Edgar Acosta López, Rodolfo Tello Saavedra, Edgar Rojas Zacarias, Humberto Bonilla, Ever Ingaruca Álvarez, Clara Espinoza Silva

**Affiliations:** 1 Facultad de Ingenierìa en Industrias Alimentarias, Universidad Nacional del Centro del Perú, Huancayo 12006, Peru; e_2015101039i@uncp.edu.pe (G.L.S.); e_2015101034b@uncp.edu.pe (M.E.V.);; 2 Grupo de Investigaciòn en Ciencia y Tecnologìa de Alimentos Funcionales, Universidad Nacional del Centro del Perú, Huancayo 12006, Peru; 3 Centro de Investigación en Nanotecnología Aplicada a la Seguridad Alimentaria y Nutricional, Universidad Nacional del Centro del Perú, Huancayo 12006, Peru

**Keywords:** collagen, ultrasound-assisted extraction (UAE), *Cavia porcellus*, by-product valorization, biomaterials

## Abstract

The by-products of *Cavia porcellus* (legs and head) were valorized for collagen extraction using ultrasound-assisted extraction (UAE). Process optimization was performed through response surface methodology (central composite design) considering amplitude, cycle, and time as factors. Samples were pretreated with NaOH and butyl alcohol, followed by acetic acid extraction under controlled sonication. The quadratic models for yield and hydroxyproline showed excellent fit (high R^2^, R^2^adj, and R^2^pred) with no significant lack of fit. The optimal conditions were identified at 100% amplitude, cycle = 1, and 27.47 min, and these were validated experimentally, yielding 28.15 ± 0.19% collagen and 4.18 ± 0.12% hydroxyproline, values that closely matched predictions. The optimal extract exhibited a hydrodynamic diameter of 599.3 nm, a ζ-potential of −61.3 mV, and a polydispersity index of 0.33, indicating a highly stable colloidal dispersion with submicron fibrils. SEM micrographs confirmed fibrillar bundles consistent with the particle size distribution, while FTIR spectra showed characteristic amide bands indicative of triple-helix preservation. These results demonstrate that UAE of guinea pig by-products produces collagen with high structural integrity and colloidal stability, highlighting its potential for food and biomaterial applications.

## 1. Introduction

Collagen is a vital structural protein in the human body, playing an essential role in maintaining the integrity and functionality of bones, muscles, and skin [1]. As the most abundant protein, collagen constitutes a significant proportion of the body’s dry weight and is indispensable for the health of connective tissues. However, its natural production declines with age, contributing to problems such as bone loss, muscle weakness, and skin aging [2,3]. Environmental factors and poor nutrition further exacerbate this decline, making collagen supplementation a widely adopted strategy to mitigate such effects [4]. Collagen hydrolysates have been shown to stimulate collagen synthesis, thereby protecting joints, improving bone density, and enhancing skin health [5]. Owing to these properties, collagen has found extensive applications in tissue engineering, clinical medicine, the food industry, packaging, cosmetics, and medical aesthetics [6,7]. Identifying alternative external sources of collagen is therefore of great importance. Among these, the guinea pig (*Cavia porcellus*), a rodent of Andean origin, is of particular interest. Traditionally consumed for its meat, it is also raised as a pet and used as an experimental animal model. Guinea pigs have been part of Andean diets since pre-Columbian times [8]. and during the COVID-19 pandemic their consumption was promoted in local contexts as beneficial for recovery, although no scientific studies have yet clarified the underlying reasons [9]. This potential may be linked to the collagen content in their skin, legs, and head, given the established role of collagen in regenerative medicine and orthopedics. Clinical studies have demonstrated that oral administration of collagen peptides significantly improves osteoarthritis symptoms, reducing WOMAC and VAS indices (*p* < 0.01) [10]. In these trials, patients receiving collagen supplements reported lower pain perception and reduced knee stiffness compared to placebo. In biomedical research, collagen-based dressings and scaffolds (gelatins, hydrogels, or matrices) are known for their low immunogenicity and high biocompatibility, which promote tissue healing and regeneration [11].

From a commercialization standpoint, guinea pig legs and heads are typically excluded from vacuum-packaged products and discarded. Within a circular economy framework, these by-products represent an underutilized resource whose valorization could reduce waste and generate value-added applications, consistent with global recommendations on sustainable livestock systems [12]. In this context, extraction methods are critical for determining collagen quality. Ultrasound-assisted extraction (UAE) has emerged as a superior alternative to conventional approaches, offering higher yields while maintaining structural integrity [13,14]. Several studies have confirmed that ultrasound improves extraction efficiency: for example, UAE increased collagen recovery from chicken meat by up to 40% compared with traditional methods [15,16]. Similarly, ultrasound treatment of lamb legs enhanced collagen yield without compromising quality [17].

Therefore, the present study aimed to evaluate the effects of amplitude, cycle, and extraction time on collagen obtained from the legs and heads of *Cavia porcellus* using ultrasound-assisted extraction.

## 2. Materials and Methods

### 2.1. Animal Material

Young male guinea pigs (*Cavia porcellus*) with an average live weight of approximately 650 g were used in this study. Following slaughter for human consumption, the carcasses were processed and the non-edible parts (legs and head) were separated. These anatomical portions, which are not normally consumed, were specifically selected as the raw material for collagen extraction in this work, whereas the remaining carcass was intended for human consumption.

### 2.2. Extraction Method

Samples from guinea pig legs and heads were pretreated with 0.1 M NaOH solution at a 1:10 (*w*/*v*) ratio [15] for 24 h to remove non-collagenous proteins. After pretreatment, the samples were rinsed with distilled water until a neutral pH (≈7.0) was reached, followed by treatment with 10% butyl alcohol for 24 h to remove lipids. They were then washed again with distilled water. Subsequently, the samples were immersed in 0.05 M acetic acid (1:30, *w*/*v*; skin and muscle from legs and head: acetic acid) and ultrasonicated using a Hielscher UP100H ultrasonic processor (100 W, 30 kHz, probe-type sonotrode) under the conditions specified in the experimental design. The temperature was maintained between 5–7 °C using an ice bath. Following ultrasonication, the samples were stirred magnetically for 48 h to facilitate extraction.

A control extraction without ultrasonication was also performed following the same procedure, except that the ultrasonic step was omitted. After pretreatment with NaOH, the control samples were washed, immersed in butyl alcohol, rinsed, and stirred for 48 h. Both UAE and control extracts were filtered and freeze-dried to determine the extraction yield. The final moisture content of the dried samples was approximately 6%.

### 2.3. Experimental Design

Response surface methodology (RSM) was applied using a Central Composite Design (CCD) to optimize the extraction process. The factors considered were amplitude, cycle, and time, with their levels determined from a comprehensive review of the literature [15] and preliminary experimental trials. A detailed description of the independent variables used in the design is presented in Table 1.

### 2.4. Collagen Extraction Yield

The yield of collagen extraction was calculated from the ratio between the mass of the fresh sample and the mass of the freeze-dried collagen [17] according to the following equation:

R (%) = M_Collagen_/M_sample_ × 100 (1)

where:

R: collagen extraction yield (%)
M_Collagen_: Collagen mass obtained (g)
M_sample_: Initial guinea pig head and leg mass (g).

### 2.5. Quantification of Hydroxyproline

The spectrophotometric method described by [16] was used to quantify hydroxyproline. Briefly, 1 g of lyophilized collagen was hydrolyzed with 1 mL of 6 N HCl at 130 °C for 3 h in an autoclave. The hydrolysate was diluted to volume in a 250 mL volumetric flask, after which 0.02% methyl red indicator was added, and the solution was neutralized with 2.5 N NaOH until the indicator turned slightly yellow (pH 6–7). From this solution, 0.1 mL was transferred into a 16 × 150 mm test tube, followed by the addition of 0.9 mL of 0.2 M NaCl and 1 mL of chloramine T. The mixture was thoroughly shaken and allowed to stand for 20 min at room temperature. Subsequently, 1 mL of perchloric acid was added to degrade the chloramine T, mixed, and left to stand for 5 min. Thereafter, 1 mL of p-dimethylaminobenzaldehyde solution was added, and the mixture was vigorously shaken until homogeneous. The tube was placed in a water bath at 60 °C for 20 min and then cooled under running water for 5 min. The developed color remained stable for at least 1 h. Absorbance was measured at 557 nm using a spectrophotometer.

Hydroxyproline concentration was determined using a standard calibration curve, expressed by the following regression equation:

Y = 0.0998X + 0.0253
(2)

R^2^ = 0.9987

where:

Y: Hydroxyproline content (μg/mL)
X: Absorbance (nm).

### 2.6. Determination of ζ-Potential by Dynamic Light Scattering (DLS)

The ζ-potential and polydispersity index (PDI) were measured using a Brookhaven DLS model 90Plus analyzer (Brookhaven Instruments Corporation, Nashua, NH, USA) equipped with dedicated software for ζ-potential determination. Approximately 10 mg of particles were dispersed in 100 mL of ultrapure water. The samples were prepared in KCl solution, stirred, and sonicated to ensure homogeneous dispersion. Measurements were conducted at 25 °C using a 658 nm laser, with an analysis time of 1.5 min.

### 2.7. Particle Morphology and Size Analysis by SEM

The microstructures of tracheal tissues pretreated with alkali and ultrasound were analyzed using a scanning electron microscope (JSM-6010LV, JEOL Ltd., Tokyo, Japan). Collagen samples extracted from guinea pig legs and heads were examined with a Quanta 450 scanning electron microscope (FEI Co., Hillsboro, OR, USA). All specimens were mounted on aluminum stubs with double-sided conductive adhesive tape and coated with gold using a sputter coater (JFC-1100E, JEOL Ltd., Tokyo, Japan) for 1 min. Microstructural features were observed at an accelerating voltage of 15 kV and a magnification of 500×.

### 2.8. Fourier Transform Infrared Spectroscopy (FTIR)

The structural characteristics of collagen were analyzed by Fourier Transform Infrared Spectroscopy (FTIR) using a Perkin Elmer FTIR Spectrometer (Perkin Elmer, Springfield, IL, USA). Lyophilized collagen samples were ground into a fine powder and analyzed directly using the ATR (attenuated total reflectance) accessory. Spectra were recorded in the range of 4000–400 cm^−1^ with a resolution of 4 cm^−1^ and an average of 32 scans per sample. The characteristic absorption bands (Amide A, Amide I, Amide II, and Amide III) were identified to evaluate the preservation of the collagen triple-helix structure.

### 2.9. Statistical Analysis

A 2ⁿ factorial design was employed to evaluate the effects of three independent variables: X_1_ (amplitude), X_2_ (cycle), and X_3_ (time) on the extraction of freeze-dried collagen from guinea pig legs and heads. The response variables were collagen yield (lyophilized) and hydroxyproline content.

Statistical analyses were performed using Design-Expert software, version 11 (Stat-Ease Inc., Minneapolis, MN, USA). The experimental data were analyzed through multiple regression using the least squares method. Regression coefficients for all linear, quadratic, and interaction terms were evaluated by analysis of variance (ANOVA). Model significance was assessed with Fisher’s F-test at a confidence level of *p* ≤ 0.05.

The adequacy of the models was judged using the coefficient of determination (R^2^), the predicted coefficient of determination (R^2^pred), and the adjusted coefficient of determination (R^2^adj). Once validated, response surface plots and contour graphs were constructed to describe and predict the relationships between the independent and response variables.

## 3. Results

### 3.1. Effect of Variables on Yield and Hydroxyproline

Table 2 presents the experimental results generated by the software. The data were analyzed, and model fitting was assessed, showing that the quadratic model provided the best fit. The coefficients of determination (R^2^) for collagen yield and hydroxyproline content were 0.9971 and 0.9976, respectively. The adjusted R^2^ values were 0.9933 for yield and 0.9946 for hydroxyproline, while the predicted R^2^ values were 0.9710 and 0.9782, respectively.

The evaluation of model adequacy confirmed that the quadratic model was the most statistically significant compared with the other models tested. This result indicates that at least one of the regression terms showed a significant correlation within the central composite design (CCD).

#### 3.1.1. Model Fitting and Statistical Analysis by Response Surface Methodology (RSM)

A central composite design (CCD) with three factors at three levels was applied to optimize the combined effects of amplitude (X_1_), cycle (X_2_), and time (X_3_) on extraction yield (%) and hydroxyproline content (%). A total of 17 experimental runs were carried out to evaluate the influence of these variables (Table 2). The mathematical model was developed using multiple regression analysis to describe the interactions of the independent variables on both responses. The corresponding second-order polynomial equation was expressed as follows:

For Collagen yield:

Yield = +9.28 + 3.51 X_1_ + 4.89 X_2_ + 5.48 X_3_ + 2.76 X_1_X_2_ − 0.7763 X_1_X_3_
− 0.1238 X_2_X_3_ − 0.2493 X_1_^2^ + 5.7507 X_2_^2^ − 1.0042 X_3_^2^ (3)

For hydroxyproline content:

Hydroxyproline content = + 3.118 + 0.5372 X_1_ +
0.5372 X_2_ + 0.8408 X_3_ + 0.1147 X_1_X_2_
+ 0.0670 X_1_X_3_ + 0.1302 X_2_X_3_ + 1.4619
X_1_^2^ − 1.683 X_2_^2^ − 1.098 X_3_^2^ (4)

Analysis of variance of the parameters in the regression equation, for yield and hydroxyproline content.

The analysis of variance (ANOVA) results are presented in Table 3. The F-values were 2.6620 for yield and 329.49 for hydroxyproline content, with *p*-values < 0.0001 in both cases. These results indicate that the regression model was statistically significant for both hydroxyproline content and collagen yield.

The lack-of-fit test yielded a *p*-value of 0.0513 and an F-value of 18.78, confirming the high accuracy of the model. The linear coefficients (X_1_, X_2_, and X_3_) were statistically significant (*p* < 0.05), indicating that amplitude, cycle, and time were influential factors. For yield, the strongest linear effect was observed for time (X_3_), followed by cycle (X_2_) and amplitude (X_1_), with F-values of 860.7, 685.7, and 353.0, respectively (*p* < 0.0001). For hydroxyproline content, time also showed the greatest influence (F = 726.6), followed by amplitude and cycle (F ≈ 296.6 and 264.8; *p* < 0.0001).

These trends are consistent with cavitation physics, where a higher acoustic dose—defined as the product of amplitude, cycle, and time—enhances the disruption of the collagen matrix and mass transfer. Nevertheless, beyond a certain threshold, degradation and thermal denaturation effects occur [18]. In the ANOVA, the quadratic terms X_2_^2^ and X_3_^2^ were statistically significant for yield. For hydroxyproline content, the quadratic terms X_1_^2^, X_2_^2^, and X_3_^2^ were also significant, with negative coefficients for cycle and time, indicating reduced yield at higher levels. Overall, the model exhibited sufficient accuracy and reproducibility to predict collagen yield and hydroxyproline content under the conditions tested for ultrasound-assisted extraction. The model fit was exceptional, with the quadratic model accounting for nearly all the variability in both yield and hydroxyproline content (R^2^ = 0.9971 and 0.9976; R^2^_adj = 0.9933 and 0.9946; R^2^_pred = 0.9710 and 0.9782). This finding indicates that there was no significant lack of fit, thereby supporting the model’s predictive capability under the conditions examined [19].

#### 3.1.2. Interaction Effects of Process Parameters on Yield and Hydroxyproline Content in Ultrasound-Assisted Extraction

**Figure 1:**
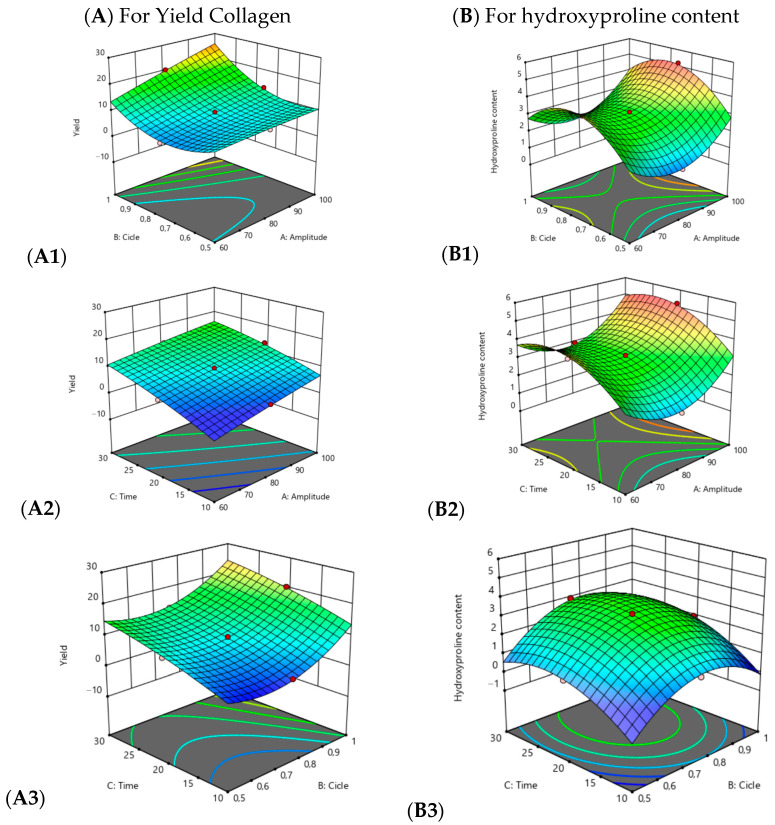
Response surface plots illustrating the interaction effects of process parameters on collagen yield and hydroxyproline content obtained by ultrasound-assisted extraction of guinea pig by-products. (**A**) Collagen yield. (**B**) Hydroxyproline content. (**A1**–**A3**) Interaction effects of amplitude × cycle, amplitude × time, and cycle × time on collagen yield. (**B1**–**B3**) Interaction effects of amplitude × cycle, amplitude × time, and cycle × time on hydroxyproline content. The pink dots ( 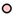) they indicate that the design points are below the predicted value, the red dots ( 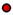) indicate that the design points are above the predicted value and the change from blue to red ( 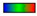) indicates the minimum and maximum value of the variables yield and hydroxyproline content respectively.

Figure 1 shows the interaction effects of amplitude, cycle, and time on collagen yield and hydroxyproline content, represented through three-dimensional response surface plots. The plots clearly illustrate how increases in these factors were associated with concomitant increases in both yield and hydroxyproline content.

The interaction between amplitude and cycle (X_1_ × X_2_) was significant for both responses, with a stronger effect observed on collagen yield, indicating a synergistic relationship between these factors. The interaction of amplitude and time (X_1_ × X_3_) significantly affected yield, whereas the interaction of cycle and time (X_2_ × X_3_) was more relevant for hydroxyproline content.

The optimal multi-response conditions (X_1_ = 100% amplitude, X_2_ = 1 cycle, X_3_ ≈ 27.47 min) yielded 28.145 ± 0.186% collagen and 4.184 ± 0.123% hydroxyproline, values that closely matched the predicted outcomes (28.718% and 4.22%, respectively). A local maximum for hydroxyproline (≈5.21%) was detected in a single run at X_1_ = 100%, X_2_ = 0.75, and X_3_ = 20 min. However, the global optimization shifted the balance toward achieving a higher overall yield with a slight reduction in hydroxyproline percentage, which is a typical result when applying the global desirability function.

#### 3.1.3. Validation of the Predictive Model

The accuracy of the predictive model was confirmed through experimental validation under the optimal conditions estimated by the response surface methodology. The model equation predicted that the optimal ultrasound-assisted extraction parameters were an amplitude of 100%, a cycle of 1, and an extraction time of 27.47 min. Under these conditions, the experimental replication yielded 28.145 ± 0.186% collagen and 4.184 ± 0.123% hydroxyproline, values that closely matched the predicted outcomes of 28.718% and 4.22%, respectively.

In addition, an extraction run was performed without using ultrasound in order to verify the importance of ultrasound and the result was 12.5%

### 3.2. Analysis of Particle Size, ζ-Potential, and Polydispersity Index (PDI)

**Figure 2:**
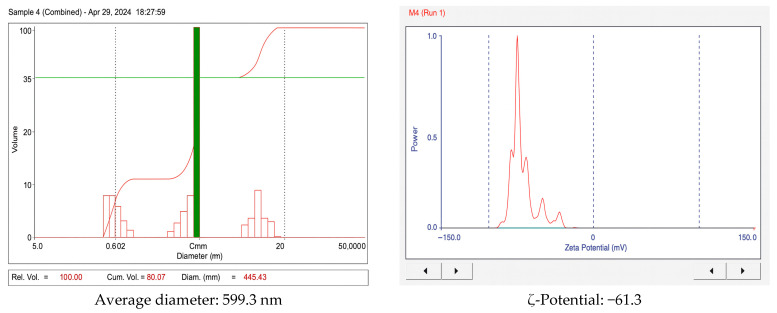
Particle size distribution and ζ-potential of collagen extracted from guinea pig heads and legs.

Figure 2 presents the particle size distribution and ζ-potential of collagen samples as determined by dynamic light scattering (DLS).

The polydispersity index (PDI) was determined to be 0.33. The average particle size of 599.3 nm suggests that the collagen particles are relatively large compared with those obtained from other animal sources. A PDI of 0.330 indicates a relatively uniform size distribution, although some variability in particle dimensions was present.

### 3.3. SEM Analysis of Surface Morphology

**Figure 3:**
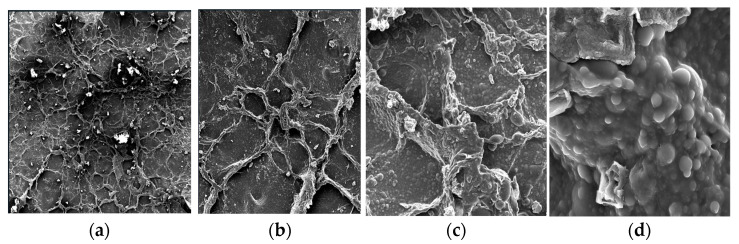
Scanning electron micrographs of collagen extracted from guinea pig legs and head at different magnifications: (**a**) 64×; (**b**) 263×; (**c**) 601×; and (**d**) 4260×.

Figure 3 displays SEM micrographs of collagen at different magnifications (64×, 263×, 601×, and 4260×), providing a detailed visualization of the surface morphology at progressively higher scales.

SEM analysis of the extracted collagen revealed fibrillar bundles with lamellar structures, likely generated during the freeze-drying process. These morphological features were in good agreement with the particle size distribution measured by DLS.

### 3.4. Fourier Transform Infrared (FTIR) Spectral Analysis

**Figure 4:**
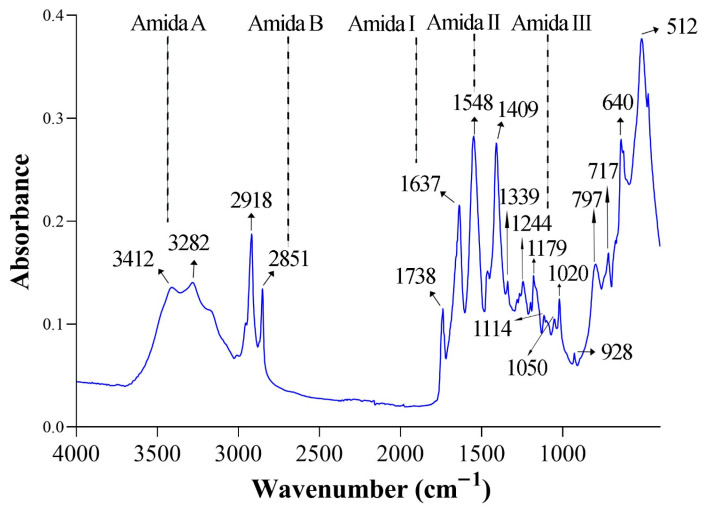
Fourier Transform Infrared (FTIR) spectra of guinea pig collagen (heads and legs), highlighting characteristic amide and functional group bands.

As shown in Figure 4, the FTIR spectra of collagen extracted from guinea pig skin by ultrasound-assisted alkaline hydrolysis exhibited the characteristic amide bands (A, B, I, II, and III), as well as absorption peaks corresponding to C–H_2_, C=O ester, C–O–C, C–O, and PO_4_^3−^ functional groups.

Table 4 summarizes the observed wavenumber positions of the collagen bands, their reference ranges reported in the literature, the corresponding vibrational assignments, and the structural significance of each absorption signal.

**Table 4:** FTIR band positions of guinea pig collagen with reference ranges, vibrational assignments, and structural significance.

Observed Band (cm^−1^)	Reference Band (cm^−1^)	Vibrational Mapping	Structural Meaning	References
3412	3307–3428	Stretching N–H (Amid A)	Hydrogen bridge network that stabilises the triple helix	[20]
3282	3282	Amid A–Streching N–H_2_	Indicates extra reinforcement of H bonds with the peptide skeleton.	[21]
2851	2924	Symmetrical stretching C–H_2_ (Amid B)	Indicates the presence of prolines and glycines in collagen.	[22]
1637	1638	Stretching C=O (Amid I)	It reflects the α/β helix conformation of the polymer and the formation of H bonds between adjacent N–H and C=O.	[23,24]
1548	1548	Flexion N–H + stretching C–N (Amid II)	Reflects peptide packaging in the triple helix.	[25,26]
1409	1410–1460	Symmetrical stretching COO^−^	Indicates the presence of deprotonated acid residues (glutamate/aspartic acid) and calcium/inorganic salts.	[27]
1339	1338	CH_2_ balance (Amid III)	Triple propeller integrity indicator.	[28]
1244	1243	Stretching C–N + flexion N–H (Amid III)	Vibrational support of the native helix	[29]
1179	1180	Stretching C–O–C	It is often associated with ether bonds in collagen/hydroxyapatite mixtures.	[30]
1020/1050	1023	Vibration PO_4_^3−^	Its presence suggests the infiltration of mineral salts or bone remains (apatite) in the sample.	[31]

The observed FTIR bands were consistent with reference ranges reported for type I collagen, confirming the presence of characteristic amide groups and functional vibrations. The identification of amide I, II, and III bands, together with amide A and B, indicates the preservation of the triple-helix structure in the extracted collagen. Minor shifts in peak positions compared with literature values may be attributed to the extraction process and the influence of ultrasound treatment, which can modify hydrogen bonding and molecular packing without fully disrupting the collagen structure. These findings support the structural integrity of the collagen obtained from guinea pig by-products and its potential applicability in biomaterial and food systems.

## 4. Discussion

The results were compared with those of other studies, such as [32], which focused on avian cartilaginous matrices. In that study, ultrasound-assisted extraction of collagen from chicken trachea was optimized, and it was reported that at an intensity of 17.46 W·cm^−2^ for 20 min, the yield doubled compared with conventional methods, without compromising the triple helix. This finding is consistent with the hypothesis that extraction times of 20–30 min combined with high amplitudes/cycles maximize yield before negative curvature occurs due to overexposure. Furthermore, it supports the idea that ultrasound-assisted extraction preserves structural integrity.

The effect of ultrasound on marine sources has also been evaluated [15]. In albacore skin (*Thunnus alalunga*), increasing ultrasonic power improved rheological and structural properties, although excessive power was reported to disrupt the fibrillar network (partial denaturation)—a phenomenon consistent with the negative quadratic terms for time and cycle observed in the present study.

At the methodological level, response surface methodology and central composite designs (CCD) are widely employed to optimize ultrasound extraction of biopolymers. For example, the optimization of gelatin extraction from buffalo skin [15], using hydroxyproline as a variable, also identified quadratic models with curvature terms and significant interactions, analogous to those found in this work. A general consensus across reviews [33] is that amplitude, time, and temperature/pH govern collagen yield and quality, and that ultrasound extraction enhances performance compared with acid or enzyme methods, while also reducing extraction times.

The finding that cycle 1 (continuous mode) was optimal contrasts with studies of ultrasound extraction from plant matrices, where pulsed modes (cycles < 1) have been shown to improve efficiency through diffusive relaxation [34]. In collagen-rich connective tissues, characterized by higher density and cross-linking, continuous mode may provide a more effective acoustic dose within the 20–30 min timeframe. This hypothesis is supported by the present results.

The use of hydroxyproline as a marker is also appropriate: the classic colorimetric method establishes a correlation between hydroxyproline percentage, collagen content, and the thermal stability of the triple helix [35]. This supports the hypothesis that an increase in hydroxyproline with higher acoustic dose may enhance collagen recovery up to a threshold, beyond which overexposure degrades the structure.

The hydroxyproline content obtained in this study (≈4.2%) is lower than that typically reported for mammalian collagens, such as bovine and porcine, which generally range between 8% and 13% of total amino acids. The higher values in mammals are associated with greater cross-link density and consequently higher thermal stability [36,37]. In contrast, marine collagens, such as those from fish skin, usually contain ≈5–7% hydroxyproline, which explains their lower denaturation temperatures and their preferential use in nutraceutical applications, where higher solubility and bioavailability are advantageous [20,28]. The value found in guinea pig collagen lies in an intermediate range, closer to marine species than terrestrial mammals. This suggests that although guinea pig collagen may not reach the thermal resistance of bovine or porcine collagen, it provides favorable characteristics for applications in supplements and biomaterials where solubility and ease of processing are critical, thereby broadening its potential valorization as an alternative collagen source.

With respect to particle size, ζ-potential, and polydispersity index, the results indicate the presence of submicron fibrillar assemblies rather than individual tropocollagen molecules (a triple helix measures approximately 300 nm × 1.5 nm) [38], The high magnitude of ζ-potential (−61.3 mV) reflects excellent electrostatic stability due to strong surface charge repulsion [20], while the polydispersity index of 0.33 indicates a moderately broad but biologically acceptable size distribution [28]. These findings are consistent with the principles of DLS physics, in which the hydrodynamic diameter encompasses the solvation layer [39].

The average hydrodynamic diameter obtained (≈599 nm) suggests the presence of fibrillar assemblies rather than isolated tropocollagen. From a technological perspective, this particle size may be advantageous for food and biomedical applications. In food matrices, submicron fibrils can enhance water-holding capacity and viscosity, improving texture and gel formation, as observed in fish and poultry collagens [28,33]. In biomedical contexts, submicron fibrillar aggregates provide a framework closer to native extracellular matrices, promoting cell adhesion and tissue regeneration [40]. However, compared with soluble collagen peptides, the relatively large size could limit direct nutraceutical use, since smaller peptides (<10 kDa) are generally preferred for higher bioavailability [5]. Thus, while the observed particle size confers stability and functional potential in biomaterials, additional hydrolysis or fractionation may be necessary to tailor its suitability for oral supplementation.

In colloid science, ζ-potential values of at least |30| mV are generally regarded as indicative of “good” stability, while values ≥ |60| mV reflect “excellent” stability, due to the strong electrostatic repulsion that minimizes aggregation. The observed value of −61.3 mV confirms the outstanding stability of guinea pig collagen dispersions, consistent with previous reports for collagen from Acaudina molpadioides, where highly negative ζ-potential values were associated with minimal aggregation [41]. Comparative studies on bovine, porcine, and fish-derived gelatins have also demonstrated that more negative ζ-potentials correlate with higher stability, reinforcing the interpretation that guinea pig collagen exhibits excellent colloidal integrity [42].

SEM analysis corroborated these findings, revealing fibrillar bundles and lamellar textures consistent with self-assembled structures typically observed after drying. While DLS analysis reflects hydrated fibrils in dispersion, SEM preparation (dehydration and coating) can induce collapse and wrinkling, leading to the appearance of dense textures despite high stability in solution [15,42]. Similarly, Amide II (~1548 cm^−1^) and Amide III (~1244 cm^−1^) bands corroborated the presence of secondary structure. Additional peaks, such as COO^−^ symmetric vibrations (~1409 cm^−1^) and C–O–C stretching (~1179 cm^−1^), aligned with values reported for type I collagen, while the PO_4_^3−^ signal at 1020–1050 cm^−1^ may reflect residual hydroxyapatite [30,31]. These findings confirm that ultrasound-assisted acetic acid extraction preserved the triple helix more effectively than conventional acid-based methods, which often show peak shifts and band broadening due to partial denaturation [25,26].

Guinea pig collagen presents distinctive advantages compared with traditional sources. Nutritionally, its amino acid profile—with intermediate levels of hydroxyproline and proline—resembles marine collagens, favoring solubility and bioavailability in nutraceutical applications [5,20]. From a production standpoint, guinea pigs are small animals with rapid growth rates and high feed conversion efficiency compared with bovine and porcine species, making them a sustainable and low-impact source aligned with circular economy principles [12]. Additionally, guinea pig husbandry is a traditional Andean practice, ensuring cultural acceptance and facilitating the valorization of by-products such as heads and feet that are otherwise discarded. Technologically, guinea pig collagen demonstrated excellent colloidal stability (ζ-potential ≈ −61 mV) and submicron fibrillar assemblies, attributes that are valuable for functional foods, biodegradable packaging, and biomaterials.

Beyond its biochemical and technological attributes, guinea pig collagen also embodies sustainability and cultural value. From a sustainability perspective, guinea pigs require less land, water, and feed than large livestock species, and their short reproductive cycles enable efficient protein production with a reduced environmental footprint. Valorizing by-products such as heads and feet directly supports circular economy strategies, reducing waste and enhancing small-scale production efficiency [12]. From a cultural perspective, guinea pig husbandry is deeply rooted in Andean traditions, where the species has nutritional, economic, and symbolic significance. Incorporating cultural acceptance into the valorization process not only facilitates consumer uptake but also empowers local communities by generating value-added products from underutilized resources. Taken together, guinea pig collagen represents not only a functional biomaterial but also a sustainable and culturally embedded alternative, reinforcing its relevance in the broader framework of by-product valorization.

## 5. Conclusions

The findings of this study demonstrated that optimization of amplitude, cycle, and time using RSM/CCD generated robust quadratic models for yield and hydroxyproline content, with no significant lack of fit, confirming the predictive reliability of the models within the experimental region. ANOVA analysis showed that all three ultrasonic parameters exerted significant effects on extraction outcomes.

Colloidal characterization of the optimal sample by DLS revealed highly stable electrostatic dispersions (ζ ≈ −61 mV) with submicron fibrillar assemblies rather than individual tropocollagen molecules, consistent with moderate polydispersity (PDI ≈ 0.33). SEM micrographs corroborated these findings, displaying fibrillar and lamellar bundles that aligned with the size distribution obtained by DLS. FTIR spectra exhibited the characteristic amide bands (Amide I, II, and III), confirming preservation of the triple helix and indicating that no significant denaturation occurred under the optimized conditions.

Overall, ultrasound-assisted extraction of collagen from guinea pig legs and heads was shown to be an effective strategy for the valorization of animal by-products, achieving high yields while preserving structural integrity. The resulting colloidal stability and fibrillar morphology confer advantages for applications in food systems and biomaterials. Future work should explore process scaling, fine thermal control during UAE, and complementary validations (SDS-PAGE, DSC, rheology) to maximize functional performance and broaden potential applications.

## Figures and Tables

**Table 1:** Independent variables and levels used in the Central Composite Design (CCD) for ultrasound-assisted collagen extraction from guinea pig by-products.

Factor	Code	Levels		
		−1	0	1
Amplitude (%)	X_1_	60	80	100
Cycle	X_2_	0.5	0.75	1
Time (min)	X_3_	10	20	30

The data were entered into the software to generate the experimental treatments.

**Table 2:** Results of the CCD analysis on yield and hydroxyproline content.

Run Numbers	Amplitude (X_1_)	Cycle (X_2_)	Time (X_3_)	Yield (%)	Hydroxyproline Content (%)
1	60	1	30	19.03	2.531
2	60	0.5	30	14.61	1.552
3	100	1	30	29.11	3.971
4	60	0.75	20	4.81	3.921
5	60	1	10	6.11	0.831
6	80	0.75	20	9.23	3.111
7	100	1	10	20.12	1.891
8	80	0.75	20	9.23	3.161
9	80	0.5	20	9.71	0.891
10	80	0.75	20	9.63	3.152
11	80	1	20	20.22	1.951
12	100	0.5	30	14.82	2.421
13	100	0.5	10	4.51	0.974
14	100	0.75	20	13.12	5.211
15	60	0.5	10	2.02	0.261
16	80	0.75	10	3.21	1.061
17	80	0.75	30	13.21	2.951

**Table 3:** Analysis of variance (ANOVA) for regression parameters of collagen yield and hydroxyproline content (*p*-values < 0.0001 should be marked as ****).

Source	Sum of Squares	df	Mean Square	F-Value	*p*-Value	
**For Yiel Collagen**						
Model	836.18	9	92.91	266.20	<0.0001	****
X_1_-Amplitude	123.20	1	123.20	352.99	<0.0001	
X_2_-Cycle	239.32	1	239.32	685.68	<0.0001	
X_3_-Time	300.41	1	300.41	860.73	<0.0001	
X_1_X_2_	57.19	1	57.19	163.86	<0.0001	
X_1_X_3_	4.82	1	4.82	13.81	0.0075	
X_2_X_3_	0.1225	1	0.1225	0.3510	0.5722	
X_1_^2^	0.1665	1	0.1665	0.4771	0.5120	
X_2_^2^	88.60	1	88.60	253.87	<0.0001	
X_3_^2^	2.70	1	2.70	7.74	0.0272	
Residual	2.44	7	0.3490			
Lack of Fit	2.34	5	0.4673	8.76	0.1056	not significant
Pure Error	0.1067	2	0.0533			
Cor Total	838.62	16				
**For hydroxyproline content**						
Model	28.85	9	3.21	329.49	<0.0001	****
X_1_-Amplitude	2.89	1	2.89	296.60	<0.0001	
X_2_-Cycle	2.58	1	2.58	264.81	<0.0001	
X_3_-Time	7.07	1	7.07	726.57	<0.0001	
X_1_X_2_	0.1053	1	0.1053	10.83	0.0133	
X_1_X_3_	0.0359	1	0.0359	3.69	0.0962	
X_2_X_3_	0.1357	1	0.1357	13.95	0.0073	
X_1_^2^	5.72	1	5.72	588.26	<0.0001	
X_2_^2^	7.59	1	7.59	780.33	<0.0001	
X_3_^2^	3.23	1	3.23	332.22	<0.0001	
Residual	0.0681	7	0.0097			
Lack of Fit	0.0667	5	0.0133	18.78	0.0513	not significant
Pure Error	0.0014	2	0.0007			
Cor Total	28.92	16				

## Data Availability

The original contributions presented in this study are included in the article. Further inquiries can be directed to the corresponding author.
